# Serum thymic stromal lymphopoietin (TSLP) levels in atopic dermatitis patients: a systematic review and meta-analysis

**DOI:** 10.1007/s10238-023-01147-5

**Published:** 2023-07-29

**Authors:** Marlenne Marisol García-Reyes, Luis Carlos Zumaya-Pérez, Rodolfo Pastelin-Palacios, Mario Adán Moreno-Eutimio

**Affiliations:** 1 Facultad de Química, Universidad Nacional Autónoma de México (UNAM), Circuito Exterior S/N, Coyoacán, Cd. Universitaria, CP 04510 Mexico City, Mexico; 2 Escuela Nacional de Ciencias Biológicas, Instituto Politécnico Nacional (IPN), Manuel Carpio, Plutarco Elías Calles, Miguel Hidalgo, CP 11350 Mexico City, Mexico

**Keywords:** TSLP, Atopic dermatitis, Eczema

## Abstract

Thymic stromal lymphopoietin (TSLP) is critical in developing allergic responses, including atopic dermatitis (AD). We systematically reviewed the literature to complete a meta-analysis to quantitatively summarize the levels of serum TSLP in AD. The study was prospectively registered in the PROSPERO database (ID = CRD42021242628). The PUBMED, SCOPUS, and Cochrane Library databases were reviewed, and original articles investigating serum TSLP in AD patients were included. Differences in TSLP levels of AD patients and controls were summarized by standardized mean differences (SMD) using a random effects model. Study quality was assessed by applying the Newcastle‒Ottawa Scale. Fourteen studies, which included 1,032 AD patients and 416 controls, were included. Meta-analysis showed that TSLP levels were significantly higher in the AD group than in the control group (SMD = 2.21, 95% CI 1.37–3.06, p < 0.001). Stratification by geographical region, age, disease severity, TSLP determination method, sample size, and study quality revealed significantly elevated TSLP levels in European AD patients (SMD = 3.48, 95% CI 1.75–5.21, p < 0.0001), adult AD patients (SMD = 4.10, 95% CI 2.00–6.21, p < 0.0001), child AD patients (SMD = 0.83, 95% CI 0.08–1.59, p = 0.031), and all severity groups with AD compared with the control group (mild: SMD = 1.15, 95% CI 0.14–2.16, p = 0.025; moderate: SMD = 2.48, 95% CI 0.33–4.62, p = 0.024; and severe: SMD = 8.28, 95% CI 4.82–11.74, p = 2.72e−6). Noticeably, adults showed higher serum TSLP levels than children with AD, and serum TSL levels increased according to AD severity. In conclusion, our meta-analysis demonstrates that circulating TSLP levels are elevated in patients with AD. Future studies are warranted to further elucidate the sources of heterogeneity.

**Supplementary Information:**

The online version contains supplementary material available at 10.1007/s10238-023-01147-5.

## Introduction

Atopic dermatitis (AD) is one of the most prevalent chronic relapsing inflammatory skin diseases. It usually develops in childhood and may persist into adulthood; less frequently, it starts in midlife or late life, and both sexes are affected [1, 2]. The prevalence and incidence of AD have increased over the past several decades [3]. Prevalences of 15–20% among children and up to 10% among adults have been reported, making AD the 15th most common nonfatal disease [4]. However, the prevalence of AD varies among races and ethnic groups [5].

AD is characterized by recurrent, pruritic, localized eczema, often with seasonal fluctuations. Many patients also have allergic asthma, allergic rhinoconjunctivitis, food allergies, and other immediate hypersensitivity (type 1) allergies [1, 2]. The clinical diagnosis of AD is based on the morphologic features and distribution of skin lesions, associated clinical signs, and a characteristic medical history [6]. A list of 23 clinical signs and symptoms of AD was published by Hanifin and Rajka in 1980 and is still used as a clinical research benchmark. AD severity can be quantitated with the Eczema Area and Severity Index (EASI), the Scoring Atopic Dermatitis (SCORAD) scale, and the Six Areas Six Sign AD (SASSAD) [7]. However, they all have high inter- and intra-observer variation.

AD pathogenesis results from complex interactions among genetic and environmental factors, skin barrier dysfunction, microbial imbalance, immune dysregulation, and environmental triggers of skin inflammation. Thymic stromal lymphopoietin (TSLP) is an IL-7–like cytokine produced by keratinocytes, although TSLP can also be produced by airway smooth muscle cells [8], human DCs [9], mast cells [10], human monocytes [9], macrophages and granulocytes [11]. TSLP exerts its biological activities by binding to a heterodimeric receptor complex consisting of the interleukin-7 receptor α chain (IL-7Rα) and the TSLP receptor chain (TSLPR) [12, 13]. TSLP is highly expressed in the epidermis of lesioned human AD skin.

TSLP can potently activate immature myeloid dendritic cells, which subsequently prime CD4 + T cells to produce allergy-promoting cytokines (such as interleukin (IL)-4, IL-5, IL-13, and TNF-α) and induce the production of TH2-attracting chemokines (CCL22 and CCL17) [10, 14]. TSLP affects several mast cell functions, including growth, survival, and mediator release [15]. Transgenic TSLP expression in keratinocytes results in AD-like skin inflammation [16]. Nevertheless, TSLP receptor-deficient mice are protected from developing allergic skin [17].

TSLP, expressed by the barrier-defective epidermis, is released into the systemic circulation [18]. Hence, several studies have focused on the relationship between serum TSLP levels and AD, concluding that TSLP levels are altered in this disease [19–29]. However, some studies showed that AD patients present similar serum TSLP levels compared to healthy controls [30–32]. Therefore, the association between TSLP levels and AD is still uncertain. To settle these controversial issues, this meta-analysis was performed to evaluate the relationship between serum TSLP levels and AD patients.

## Materials and methods

This systematic review was conducted using a prospective protocol based on the Preferred Reporting Items for Systematic Reviews and Meta-Analyses 2020 (PRISMA) statement [33]. Details of the protocol were registered on Prospero (ID = CRD42021242628). Two authors were independently involved in the study selection (LZ and MAM), data extraction (MMG and MAM), and quality assessment (LZ and MMG), and disagreements were resolved by discussion with a third author (RP) if a consensus could not be reached. In this meta-analysis, ethical approval was unnecessary, as all the data were based on previously published studies.

### Literature search strategy

We searched the PubMed, Scopus, and Cochrane Library databases for articles published from their inception until March 2021. The search strategy for each database is detailed in Supplementary Table 1. Titles and abstracts identified by the search were screened, and then full texts of selected articles were reviewed. All references cited were also reviewed to identify additional studies not indexed by the electronic databases.

### Eligibility criteria

We included any peer-reviewed English language article that examined the relationship between serum TSLP levels and AD and used a case‒control, cross-sectional, cohort, or randomized control trial (RCT) study design. Any criteria for AD diagnosis were accepted. All studies had to measure serum TSLP levels, report mean differences with standard deviation, and have sufficient data for computation. Studies were excluded if they did not contain objective data on serum TSLP levels. We recruited data only from the full-published paper, and meeting and conference abstracts were excluded.

### Data abstraction

We extracted information from each study using a predefined data extraction form designed for this review. For each eligible study, the following information was extracted: first author’s name, year of publication, journal, country, number of cases and controls with levels of TSLP measured, age, severity in the AD group, and mean and standard deviation of serum TSLP level (pg/mL). In most studies, the mean and deviation were obtained, but in several studies, only the median values and quartiles were reported. Therefore, when the original data were median values and quartiles, we transformed and calculated the data to gain the appropriate values according to the method recommended by Hozo et al. [34]. Furthermore, some studies applied WebPlotDigitizer 4.4 software to digitize and extract the data from the scatter diagrams. If important original data were unavailable in some articles, we contacted the corresponding author by e-mail to obtain further details.

### Study quality assessments

The Newcastle‒Ottawa Scale was used to assess the quality of nonrandomized studies in the review. The evaluation comprised three broad perspectives and used the following star rating system: the selection of study groups (4 stars), the comparability of the groups (2 stars), and the ascertainment of the exposure or outcome of interest (3 stars). A study was graded as low, moderate, or high quality for scores of 0–3, 4–6, and 7–9 stars, respectively.

### Statistical analysis

We conducted meta-analyses using Stata/BE, version 17 (StataCorp LP, College Station, TX). The first meta-analysis compared the mean concentrations of serum TSLP between participants with AD and those without AD. The standardized mean difference (SMD) was chosen as the summary statistic. For all analyses, we chose a random-effects model using the DerSimonian‒Laird method. To assess the degree of heterogeneity across studies, we used the I^2^ statistic and P value of the χ^2^ squared test. I^2^ values of 25%, 50%, and 75% were considered to indicate low, moderate, and high heterogeneity, respectively. To examine potential sources of heterogeneity in the meta-analysis, subgroup analysis was performed using the following variables: geographical region, age, severity of disease, TSLP-determined method, sample size, and study quality. A sensitivity test was performed to assess each study's influence on the SMD by omitting each study individually and deleting the studies with imputed data. To examine possible heterogeneities in the meta-analysis, a meta-regression analysis was performed using the following variables: publication year, mean age, proportion of males, sample size, and NOS. We used a funnel plot and Egger’s linear regression test to assess publication bias. All p values were two-sided, and the alpha was set at 0.05.

## Results

### Study selection and characteristics

**Fig. 1:**
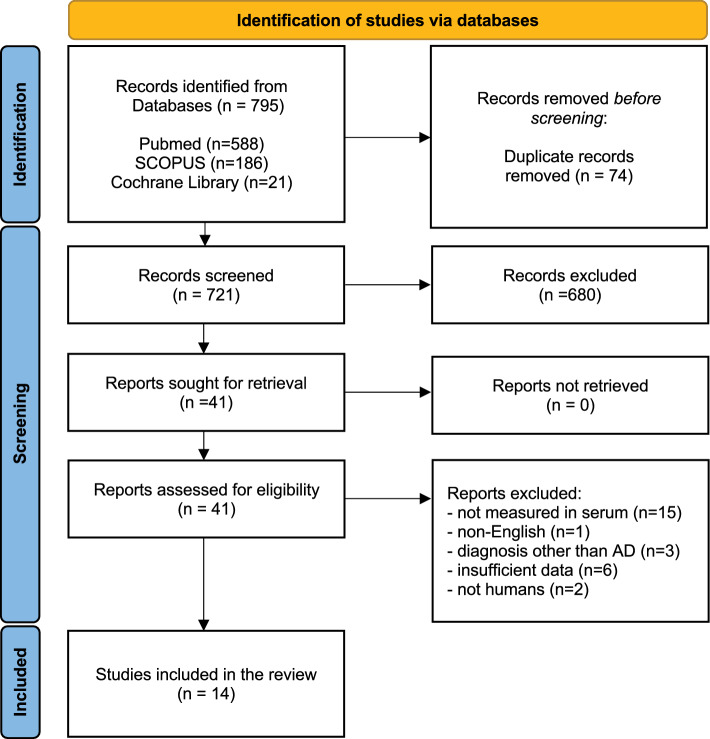
Flowchart showing article identification, inclusion, and exclusion

The initial search in the electronic PubMed, Scopus, and Cochrane Library (2002–2021) databases identified 795 studies. After screening, a total of 14 studies were included in our qualitative analysis; the results of the study selection process and reasons for exclusion are summarized in Fig. 1.

The 14 selected studies were published between 2008 and 2020 and originated from 11 different countries; seven studies were from Europe [19, 21, 23–26, 31], five studies were from Asia [22, 27, 29, 30, 32], one study was from America [28] and one study was from Africa [20]. Six studies included only children [20, 22, 26, 28–30], four studies included only adults [19, 21, 25, 32], and four studies included both children and adults [23, 24, 27, 31]. The proportion of male patients ranged from 33 to 80%, and the proportion male controls ranged from 30 to 71%. The sample sizes ranged from 9 to 165 patients and from 9 to 87 control participants (see Table 1).

**Table 1:** Characteristics of individual studies included in the meta-analysis

References	Year	Country	Sample size		Mean age (year)		Sex (M/F)		TSLP levels (pg/μL) mean ± SD			Method	Severity of AD	
			AD	Control	AD	Control	AD	Control	AD	Control			Group	Scale
Byeon et al. [29]	2020	Korea	38	10	8.4	7.9	17/21	5/5	14.20 ± 5.70	9.03 ± 4.37	ELISA		Mild Moderate	23.7 ± 10.3^a^
Jaworek et al. [21]	2020	Poland	31	20	40	37.25	15/16	10/10	80.80 ± 12.50	14.60 ± 4.50	ELISA		Severe	63.4 ± 7.3^a^
Wang et al. [27]	2020	China	55	15	12.88	NA	36/19	NA	112.74 ± 256.75	24.87 ± 30.84	Multiplex immunoassay		Moderate	33.2 ± 7.5^a^
			165	45	47.14	NA	108/57	NA	159.74 ± 638.12	49.69 ± 59.96				32.4 ± 6.7^a^
Thijs et al. [25]	2017	Netherlands	95	30	30.6	39.11	38/57	15/15	15.1 ± 1.2	3.34 ± 1.24	Multiplex immunoassay		Moderate	22.3 ± 0.33^b^
			98		31.1		43/55		17.00 ± 1.40				Severe	39.1 ± 0.57^b^
Uysal et al. [26]	2017	Turkey	60	31	1.4	1.2	30/30	16/15	193.09 ± 349.21	31.52 ± 27.11	ELISA		Mild Moderate Severe	38.6 ± 8.9^a^
Genedy et al. [20]	2016	Egypt	60	30	8.42	8.5	29/31	15/15	103.00 ± 43.28	98.50 ± 30.14	ELISA		Moderate	36.6 ± 9.6^a^
Lee et al. [30]	2016	Korea	45	21	7.54	NA	23/22	NA	9.10 ± 2.59	11.08 ± 3.94	ELISA		NA	NA
Mihaly et al. [31]	2016	Hungary	20	20	20	21	8/12	6/14	3825.00 ± 7915.68	2430.00 ± 5187.68	ELISA		NA	NA
Nygaard et al. [24]	2016	Denmark	61	31	8.4	38.7	49/12	21/10	17.30 ± 32.90	10.90 ± 30.10	ELISA		Mild Moderate Severe	35.7 ± 20.9^a^
	2016	Denmark	71	31	32.6	38.7	57/14	21/10	67.90 ± 332.40	10.90 ± 30.10	ELISA		Mild Moderate Severe	31.4 ± 18.8^a^
Mocsai et al. [23]	2015	Hungary	49	10	19	28	22/27	5/5	22.63 ± 2.60	13.87 ± 1.92	ELISA		Mild Moderate	4.85^c^
Yao et al. [28]	2013	USA	54	25	0.5–1.5	NA	NA	NA	216.32 ± 48.37	73.93 ± 24.87	Multiplex immunoassay		NA	NA
Alysandratos et al. [19]	2010	Greece	9	9	41.33	42.8	3/6	3/6	626.75 ± 751.59	15.625 ± 20.91	ELISA		Mild	NA
Lee et al. [22]	2010	Korea	75	87	5.01	4.6	72/73	52/35	29.95 ± 6.99	20.11 ± 3.94	ELISA		Mild Moderate Severe	38.7 ± 27.1^a^
Nakamura et al. [32]	2008	Japan	46	32	26.5	29.7	19/27	16/16	192.20 ± 54.10	112.60 ± 52.60	ELISA		NA	NA

*AD* atopic dermatitis, *a*: data from figure, (NA): Not available

^a^SCORAD: Severity Scoring of Atopic Dermatitis index

^b^SASSAD score

^c^TIS: Total body severity assessment

The control groups were composed of healthy individuals [19–27, 29–32] or nonatopic patients [28]. Ten of the 14 included observational studies used predefined criteria for AD: 9 studies used Hanifin and Rajka’s criteria[20–22, 24–27, 31, 32], and one study used the International Study of Asthma and Allergies in Childhood (ISAAC) questionnaire criteria [30]; the rest of the studies did not report the diagnostic criteria used for patients with AD [19, 23, 28, 29]. Eleven studies determined the TSLP concentration in serum using ELISA [19–24, 26, 29–32], and 3 used a multiplex immunoassay [25, 27, 28]. The main characteristics of the 14 observational studies included in the review are summarized in Table 1.

### Meta-analysis of serum TSLP levels in AD patients compared to controls

**Fig. 2:**
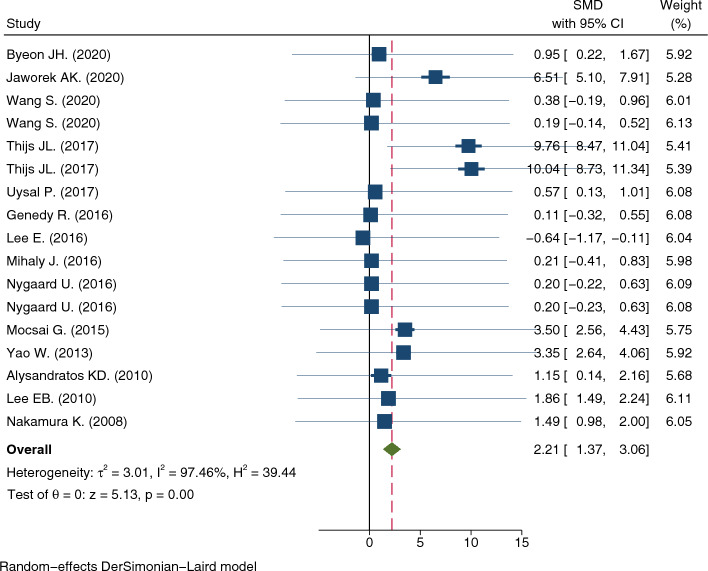
Meta-analysis of the relationship between serum TSLP levels and AD in all study subjects

**Fig. 3:**
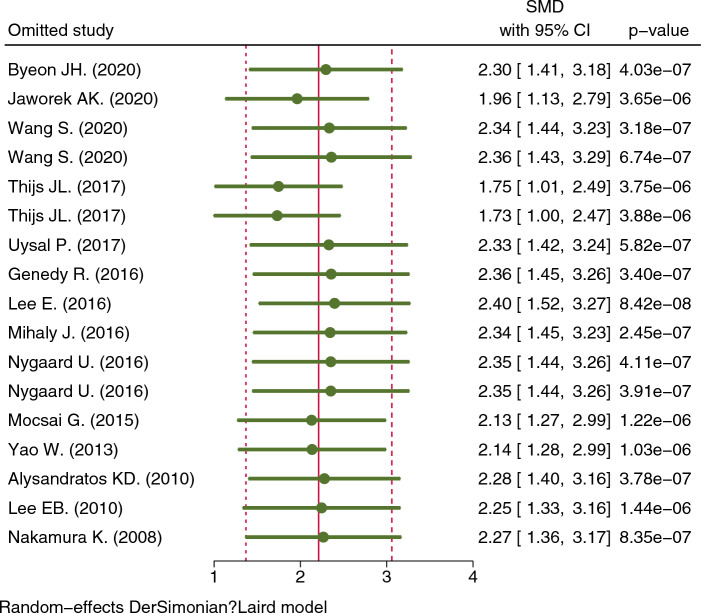
Sensitivity test of studies that examined the association between TSLP levels and AD

Of the 14 articles included in the meta-analysis, the serum TSLP levels (mean ± SD) could be extracted directly from 8 articles [19–22, 24, 25, 29, 32], whereas WebPlotDigitizer was used to digitize the graphical data from the other six articles [23, 26–28, 30, 31]. The TSLP levels were significantly higher in the AD group than in the control group (SMD = 2.21, 95% CI 1.37–3.06, p < 0.001) (Fig. 2). The total heterogeneity was very high in the meta-analysis (I^2^ = 97.46%). However, the sensitivity analysis showed that no individual study significantly affected the pooled SMD, indicating that the results of this meta-analysis were robust (Fig. 3).

The heterogeneity of each group after stratification was still very high, possibly because we measured the magnitude of heterogeneity but not the direction of effect sizes observed in the subgroups. SMDs were in the same positive direction in all of the studies, whereas there was a difference in the magnitudes of SMDs among individual studies (Fig. 2). Other unknown factors affecting heterogeneity may have contributed to the relationship between serum TSLP levels and AD.

In addition, stratification by geographical region of the studies showed significantly elevated TSLP levels in AD subjects in European populations (SMD = 3.48, 95% CI 1.75–5.21, p = 8.15e−5); in Asian populations, no difference was found. When grouped by age, children (SMD = 0.83, 95% CI 0.08–1.59, p = 3.11e−2) and adults (SMD = 4.10, 95% CI 2.00–6.21, p = 1.31e−4) showed significant differences. A greater effect on the TSLP levels was observed in adults, whereas no effect was found in the case of two studies that analyzed adults and children together Table 2.

**Table 2:** Subgroup meta-analysis of serum TSLP levels of all studies

Groups	Population	No. Of studies	Test of association			Test of heterogeneity	p value^SG^
			SMD	95% CI	*p *value	I^2^ (%)	
All		14	2.21	1.37 to 3.06	**2.90e−7**	97.46	–
Geographical region	Africa	1	0.11	**−**0.31 to 0.55	0.61	–	** < 0.001**
	America	1	3.35	2.64 to 4.06	**0.00e−10**	–	
	Asia	5	0.71	**−**0.07 to 1.49	0.076	93.92	
	European	7	3.48	1.75 to 5.21	**8.15e−5**	98.34	
Age	Adults	6	4.10	2.00 to 6.21	**1.31e−4**	98.69	**0.02**
	Children	8	0.83	0.08 to 1.59	**3.11e−2**	94.60	
	Children and adults	2	1.83	**− **1.83 to 5.06	0.265	96.96	
Severity disease	Mild	1	1.15	0.14 to 2.16	**2.51e−2**	–	** < 0.001**
	Moderate	4	2.48	0.33 to 4.62	**2.38e−2**	98.54	
	Severe	2	8.28	4.82 to 11.74	**2.72e−6**	92.32	
	Mild/Moderate	2	2.20	**− **0.30 to 4.70	0.084	94.41	
	Mild/Moderate/Severe	4	0.71	**− **0.12 to 1.54	0.092	93.73	
	NA	4	1.09	**− **0.49 to 2.68	0.177	96.60	
TSLP determination method	ELISA Assay	12	1.21	0.55 to 1.87	**3.18e−4**	94.27	**0.03**
	Multiplex immunoassay	5	4.68	1.61 to 7.76	**2.86e−3**	99.05	
Sample size	Less than 50	7	1.79	0.50 to 3.07	**6.34e−3**	95.49	0.41
	More than 50	10	2.52	1.36 to 3.67	**2.04e−5**	98.19	
Study quality	NOS < 6	9	2.06	1.20 to 2.92	**2.62e−6**	94.67	0.71
	NOS ≥ 6	8	2.39	0.90 to 3.88	**1.68e−3**	98.38	

Magnitude of standardized mean differences (SMD): 0.2–0.5, small effect; 0.5–0.8, medium effect; ≥ 0.8, large effect. pSG, the p value for differences between subgroups

In some studies, it was possible to group AD subjects by mild, moderate, and severe severity according to predefined scales, and we found higher serum levels of TSLP in the active patient groups than in the control groups for each severity level (Mild: SMD = 1.15, 95% CI 0.14–2.16; moderate: SMD = 2.48, 95% CI 0.33–4.62 and severe: SMD = 8.28, 95% CI 4.82–11.74). Interestingly, serum TSL levels increased according to the severity of AD.

The serum TSLP levels were determined by using ELISA or multiplex assays; in both cases, an effect of higher TSLP levels was observed in subjects with AD compared to in control participants (ELISA method: SMD = 1.21, 95% CI 0.55–1.87; and multiplex immunoassay method: SMD = 4.68, 95% CI 1.61–7.76). However, higher TSLP levels were observed in the studies that used the multiplex immunoassay method. The sample size of the groups in each study did not show an impact on the effect of the serum concentration of TSLP, nor did the quality of the study measured through the NOS scale.

### Metaregression analysis.

**Fig. 4:**
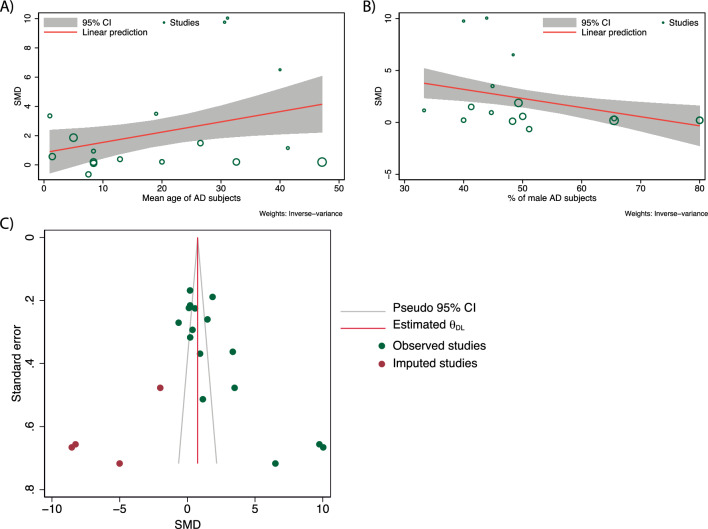
Meta-regression analysis and publication bias analysis. **A** Bubble plot for random-effects meta-regression with mean age as a study-level covariate in all AD subjects. **B** Bubble plot for random-effects meta-regression with % of male as study-level covariate on all AD subjects, the size of a bubble is in proportion to the sample size of the corresponding study. **C** The trim-and-fill method imputed six missing studies to make the funnel plot symmetrical

To investigate whether the continuous variables, including the publication year of each study, NOS score of each study, mean age, and proportion of male AD subjects, had potential moderating effects on the pooled SMD, we performed a random-effects meta-regression analysis. The meta-regression analysis showed that the mean age (p = 0.0273) and proportion of males (p = 0.0057) among AD subjects (Fig. 4A, B), but not publication year (p = 0.5313) or sample size (p = 0.1819), had a significant impact on heterogeneity in the meta-analysis of TSLP levels.

### Publication bias

The funnel plot asymmetry and Egger's regression results (P < 0.0001) indicated that there was publication bias. Therefore, the “trim and fill” method was used to adjust for bias. Compared to the previous pooled effect size (SMD = 2.21, 95% CI 1.37–3.06), the pooled SMD after adjustment (SMD = 0.762, 95% CI 0.221–1.744) remained significant, although the increased level of TSLP expression was reduced. This suggested that the meta-analysis results remained valid, although publication bias must be considered (Fig. 4C).

### Study quality assessment

Two articles were considered high quality (NOS score, 7–9) [20, 25], and 11 were considered moderate quality (NOS score, 4–6) [21–24, 26–32], thus indicating that most studies had a low risk of bias. The study quality assessment scores for observational studies are shown in Table 2. For participant selection, three studies described the representativeness of cases, one study used hospital-based controls, and two studies provided minimal or no description of where the controls were sourced. For the comparability of groups, eight studies did not match or adjust for any potential confounders [19, 21–23, 28–30, 32], and six studies matched or adjusted for only 1 factor [20, 24–27, 31]. For the ascertainment of exposure, all studies used an objective measure of TSLP determination, and the same method was used for cases and controls, but no studies provided nonresponse rates or described nonrespondents (Tables 3, 4).

**Table 3:** Statistics on meta-regression analyses regarding serum TSLP in AD

Moderator	No. of studies	Meta-regression				
		Coefficient	SE	95% CI	z	P value
The difference in the mean age	17	0.07017	0.03179	0.00786, 0.13249	2.207	**0.0273**
The difference in the proportion of male subjects	16	− 0.08773	0.03172	− 0.14990, − 0.02556	− 2.766	**0.0057**
Publication year	17	0.07814	0.12483	− 0.16653, 0.32281	0.626	0.5313
DA sample size	17	0.01789	0.01340	− 0.00837, 0.04415	1.335	0.1819
Control sample size	17	− 0.00132	0.02568	− 0.05166, 0.04902	− 0.051	0.9590
NOS	17	0.68545	0.39586	− 0.09041, 1.46131	1.732	0.0834

**Table 4:** Newcastle–Ottawa Scale (NOS) quality assessment of the studies included in the meta-analysis

References	Selection	Comparability	Exposure/outcome	Overall star rating
Byeon et al. [29]	**	*	**	5
Jaworek et al. [21]	**	*	**	5
Wang et al. [27]	**	**	**	6
Thijs et al. [25]	***	**	**	7
Uysal et al. [26]	*	**	**	5
Genedy et al. [20]	***	**	**	7
Lee et al. [30]	***	*	**	6
Mihaly et al. [31]	**	**	**	6
Nygaard et al. [24]	**	**	**	6
Mocsai et al. [23]	*	*	**	4
Yao et al. [28]	*	*	**	4
Alysandratos et al. [19]	–	*	**	3
Lee et al. [22]	**	*	**	5
Nakamura et al. [32]	**	*	**	5

Stars in the table represent study quality as per the Newcastle-Ottawa scale: selection (up to 4 stars), comparability (up to 2 stars), and exposure/outcome (up to 3 stars)

## Discussion

TSLP is a promising therapeutic target that plays a critical role in the pathogenesis of AD; therefore, in this systematic review and meta-analysis, we set out to clarify whether serum TSLP in AD patients differs from controls by analyzing the published data available to date [35]. Our meta-analysis showed that serum TSLP levels are elevated in subjects with AD and are higher in adults than in children. In addition, higher serum levels were found in studies with subjects with severe AD and in the European population, in agreement with recent research advancements indicating that AD is a complex disease characterized by different subtypes/phenotypes based on age, disease chronicity, and ethnicity [36]. Interestingly, the Asian population with AD does not present elevated levels of TSLP (SMD = 0.71, 95% CI − 0.07 to 1.49, p = 0.076), which could be partly related to the fact that the Asian AD phenotype presents a blended phenotype between that of European-American patients with AD and those with psoriasis; furthermore, this population shows increased epidermal hyperplasia, greater TH17/TH22 and lower TH1 skewing, and comparable TH2 activation [37, 38].

Our analysis showed that children with AD have lower levels of serum TSLP than adults with AD. This could be explained because adults with AD have an increased frequency of IL-22–producing CD4 and CD8 T cells within the skin-homing population compared with children, [39] and these cells are involved in chronic changes in epidermal hyperplasia, which is primarily observed in adults [40].

The current study is the first meta-analysis to clarify alterations involving serum TSLP in AD patients. However, this study had certain limitations. First, substantial heterogeneity among the studies included in this meta-analysis should be noted. We used meta-regression to compare serum TSLP levels between AD patients and healthy controls to explore whether the source of heterogeneity was derived from sex, age, sample size, year of publication, and disease activity; the meta-regression showed that age and sample size affected between-study variation. However, no statistical significance was found with sample size, year of publication, and disease activity (p value of meta-regression > 0.05). However, the conclusion that there is no relationship between the above factors and inherently high heterogeneity cannot be drawn arbitrarily because of the lack of data regarding disease activity and duration in the included studies. Although the source of heterogeneity was difficult to determine, the ethnicity, treatment, and other factors may have affected the heterogeneity of the included studies.

Due to the lack of data, we did not analyze the correlation between TSLP levels and other disease parameters, such as the severity index according to the objective SCORAD index, total serum IgE levels, serum cytokines, or chemokines. Owing to these limitations, the results of this meta-analysis should be interpreted carefully.

In addition to strategies aimed at neutralizing the functions of TSLP in Atopic Dermatitis (AD) [41], recent research has pursued promising new therapeutic avenues, explored in preclinical trials using murine models. Among these, the topical application of calcitriol, an active form of Vitamin D, has the potential to ameliorate AD symptoms by restoring the dysfunctional epidermal and tight junction barriers frequently associated with this condition [42]. Additionally, the antimicrobial peptide derived from Insulin-like Growth Factor-Binding Protein 5 (AMP-IBP5) has demonstrated potential in modulating the cutaneous inflammatory environment [43]. This spectrum of emerging therapeutic options emphasizes the vital role of ongoing research in AD and highlights the necessity for a comprehensive approach that addresses not only the symptoms but also the underlying causes of this intricate disease.

Tezepelumab is a human monoclonal antibody that targets circulating TSLP. A clinical trial showed that patients with moderate to severe AD achieved numerical improvements over placebo when treated with tezepelumab; however, these improvements were not statistically significant [41]. However, two isoforms of TSLP, short and long isoforms, have been described; the main isoform expressed during steady-state conditions is the short form of TSLP, whereas the long form of TSLP is upregulated in inflammatory conditions [44]. However, since the expression patterns and biological properties of these two different isoforms of TSLP seem to be distinct, these two TSLP isoforms should be analyzed separately in future studies. This is highlighted by findings in asthma research, where it was observed that the asthma-associated long TSLP isoform negatively regulates the secretion of IgA, potentially impacting the surveillance of mucosal surfaces detrimentally in this condition [45].

## Conclusion

Our meta-analysis demonstrates that serum TSLP levels are high in AD patients compared with non-AD subjects. Additionally, serum TSLP levels in AD adults are higher than those in AD children and increase according to AD severity. Further studies are necessary to elucidate how TSLP directly contributes to the pathogenesis of AD.

### Supplementary Information

Below is the link to the electronic supplementary material.

Supplementary file1 (DOCX 26 kb)

## Data Availability

The raw materials can be requested by communication with corresponding author.
