# Supplementary material for: Serum thymic stromal lymphopoietin (TSLP) levels in atopic dermatitis patients: a systematic review and meta-analysis
Source: Clin Exp Med. 2023 Jul 29;23(8):4129–39. doi: 10.1007/s10238-023-01147-5 (PMC10725349; doi:10.1007/s10238-023-01147-5)
Supplement: Supplementary file 1 — Supplementary file1 (DOCX 26 kb) [file 10238_2023_1147_MOESM1_ESM.docx]

**Supplementary table 1. Systematic search strategy**

A search performed on PubMed, Scopus, and the Cochrane Library databases

| 1. **PubMed:**   (“Atopic Dermatitis" OR "Dermatitis" OR "Eczema") AND ("Thymic Stromal Lymphopoietin" OR "TSLP") |
| --- |
| 1. **SCOPUS:**   TITLE-ABS-KEY (("Atopic Dermatitis" OR dermatitis OR eczema) AND ("Thymic Stromal Lymphopoietin" OR TSLP)) AND NOT (INDEX (medline)) |
| 1. **Cochrane library:**   (Atopic Dermatitis OR Eczema) AND (“Thymic Stromal Lymphopoietin” OR TSLP) in Title Abstract Keyword - (Word variations have been searched) |
